# The influence of fear of hypoglycemia and frailty on self-efficacy in diabetes management among older adults with type 2 diabetes: a cross-sectional study

**DOI:** 10.3389/fpsyg.2026.1714323

**Published:** 2026-03-11

**Authors:** Shaimaa Mohamed Amin, Haitham Mokhtar Mohamed Abdallah, Mohamed Ali Zoromba, Esteer Ibrahim Ghayth, Sameer A. Alkubati, Nada Alqarawi, Mohamed Hussein Ramadan Atta, Ibrahim Alasqah, Nesreen AbdelMonaem AbdelSataar AbouZeid, Sally Mohammed Farghaly Abdelaliem, Suebsarn Ruksakulpiwat

**Affiliations:** 1 Department of Community, Psychiatric, and Mental Health Nursing, College of Nursing, Qassim University, Buraydah, Saudi Arabia; 2 Nursing Services Department, Qassim University Medical City, Buraydah, Saudi Arabia; 3 Community Health Nursing Department, Faculty of Nursing, Damanhour University, Damanhour, Egypt; 4 College of Nursing, Jouf University, Sakaka, Saudi Arabia; 5 Nursing Department, College of Nursing, Prince Sattam Bin Abdulaziz University, Al-Kharj, Saudi Arabia; 6 Psychiatric and Mental Health Nursing, Faculty of Nursing, Mansoura University, Mansoura, Egypt; 7 Geriatric Nursing Department, Faculty of Nursing, Sohag University, Sohag, Egypt; 8 Department of Medical-Surgical Nursing, College of Nursing, University of Ha’il, Ha’il, Saudi Arabia; 9 Nursing Department, College of Applied Medical Sciences, Prince Sattam Bin Abdulaziz University, Wadi Ad-Dawasir, Saudi Arabia; 10 Psychiatric and Mental Health Nursing Department, Faculty of Nursing, Alexandria University, Alexandria, Egypt; 11 School of Health, University of New England, Armidale, NSW, Australia; 12 Department of Medical and Surgical Nursing, College of Nursing, Princess Nourah bint Abdulrahman University, Riyadh, Saudi Arabia; 13 Department of Nursing, Faculty of Allied Health Sciences, Kuwait University, Kuwait City, Kuwait; 14 Department of Nursing Administration, Faculty of Nursing, Alexandria University, Alexandria, Egypt; 15 Department of Medical Nursing, Faculty of Nursing, Mahidol University, Bangkok, Thailand

**Keywords:** diabetes mellitus, cross-sectional study, older adults, hypoglycemia phobia, frailty, self-efficacy, management, community empowerment

## Abstract

**Introduction:**

Managing type 2 diabetes mellitus (T2DM) in older adults is challenging, particularly when complicated by fear of hypoglycemia and frailty, both of which can undermine self-efficacy, a key determinant of effective disease control.

**Aim:**

To examine the influence of fear of hypoglycemia and frailty on self-efficacy in diabetes management among older adults with T2DM.

**Methods:**

A cross-sectional study was conducted at diabetic clinics, with 300 adults aged ≥ 60 years using convenience sampling. Validated tools included the Fear of Hypoglycemia Screener, Chinese Frailty Screening Scale, and Diabetes Management Self-Efficacy Scale.

**Results:**

Mean scores for fear of hypoglycemia, frailty, and self-efficacy were 36.13 ± 7.70, 12.93 ± 2.28, and 19.95 ± 3.33, respectively. Fear of hypoglycemia correlated positively with frailty (*r* = 0.277, *p* < 0.01) and negatively with self-efficacy (*r* = −0.270, *p* < 0.01). Frailty also showed a moderate negative correlation with self-efficacy (*r* = −0.454, *p* < 0.01). Regression analysis identified higher fear of hypoglycemia and frailty scores predicted lower self-efficacy (*p* < 0.05).

**Conclusion:**

Interventions addressing fear of hypoglycemia and frailty are essential to enhance self-efficacy and improve diabetes self-management in older adults.

## Introduction

Diabetes is a chronic condition resulting from insufficient insulin production or ineffective insulin utilization, leading to hyperglycemia that can damage nerves, blood vessels, and multiple organ systems (Fariba et al., 2024). In 2021, diabetes directly caused 1.6 million deaths, 53% among those aged over 60, and contributed to 530,000 deaths from kidney disease and 11% of cardiovascular deaths (Study, 2024). Although overall global mortality from the four major noncommunicable diseases (cardiovascular diseases, cancer, chronic respiratory diseases, and diabetes) has declined, this reduction has not been uniform across disease categories. In contrast to the downward trends observed for cardiovascular disease, cancer, and chronic respiratory diseases, mortality attributable to diabetes has continued to rise. This distinction clarifies that diabetes is an exception to the general decline in NCD-related mortality. Supporting this concern, the Global Burden of Disease Collaborative Network (2024) reported that in 2021, diabetes and diabetes-related kidney disease accounted for over 2 million deaths worldwide. Additionally, approximately 11% of global cardiovascular deaths were attributable to elevated blood glucose levels, underscoring the growing and interconnected burden of diabetes on global mortality.

Over a quarter of adults aged 65 years and older have diabetes, and nearly half have prediabetes, with prevalence projected to rise (Laiteerapong and Huang, 2018; Prevention, 2020). Type 2 diabetes is most common, though advances in insulin delivery have enabled people with type 1 diabetes to live well into older age. Effective management in older adults requires comprehensive medical, psychological, functional, and social assessments; accurate classification of diabetes type; evaluation of disease duration and complications; and tailored treatment that considers hypoglycemia risk (Academies, 2015; Kirkman et al., 2012; Young-Hyman et al., 2016).

Older adults with diabetes are at higher risk for functional disability, sarcopenia, hypertension, chronic kidney disease, cardiovascular disease, stroke, and premature death. They are also more prone to geriatric syndromes, including cognitive decline, depression, urinary incontinence, falls, chronic pain, frailty, and polypharmacy, which can hinder self-management and reduce quality of life if unaddressed (Laiteerapong and Huang, 2018; Laiteerapong et al., 2011; Sudore et al., 2012). Diabetes self-management, covering medication adherence, diet, exercise, glucose monitoring, and foot care, is as important as pharmacologic therapy. Education programs play a central role in promoting these behaviors (10–12), but many patients struggle to maintain them (Tahmasebi and Noroozi, 2012).

Self-efficacy, defined as an individual’s belief in their ability to perform health-promoting behaviors (Bandura, 1986), is a key determinant of diabetes self-management and is linked to better clinical outcomes and well-being (Al-Khawaldeh et al., 2012; Çalli, 2021; Rose et al., 2009). For example, Çalli (2021) identified self-efficacy and regular exercise as significant predictors of well-being in people with type 2 diabetes, with self-efficacy emerging as the strongest contributor (Çalli, 2021). Conversely, Al-Khawaldeh et al. (2012) reported that most participants had low self-efficacy and inadequate self-management behaviors (Al-Khawaldeh et al. 2012), underscoring the need to integrate self-efficacy enhancement into diabetes education. Hypoglycemia, defined as blood glucose <3.9 mmol/L (70 mg/dL) (Araszkiewicz et al., 2021), is a common complication of insulin and sulfonylurea therapy. Fear of hypoglycemia, arising from past episodes or perceived risk, is associated with reduced quality of life, treatment avoidance, and poorer glycemic control (American Diabetes Association, 2023). In people with type 1 diabetes, this fear is heightened among those with severe or recurrent episodes, particularly with impaired hypoglycemia awareness (Anderbro et al., 2015). Among individuals with type 2 diabetes, fear is more pronounced in insulin users than in those using sulfonylureas (Huang et al., 2021), and can be a barrier to insulin initiation (Huang et al., 2021; Melson et al., 2024). While some fear is adaptive, excessive fear may impair self-management and limit treatment efficacy (Martyn-Nemeth et al., 2016).

Older adults are especially vulnerable to hypoglycemia but may present with atypical or nonspecific symptoms such as dizziness or confusion, complicating diagnosis and leading to underreporting (Amiri et al., 2015; Araszkiewicz et al., 2021; Bonds et al., 2012; Cryer, 2005; Graveling and Frier, 2010a). Symptom recognition is further impaired by aging-related changes, such as lower glucose thresholds for autonomic symptoms and earlier onset of cognitive impairment during episodes, contributing to a higher prevalence of unrecognized or subclinical hypoglycemia (Cryer, 2005; Graveling and Frier, 2010a). Frailty, defined by phenotypes such as weight loss, weakness, exhaustion, slow gait, and reduced activity (Randväli et al., 2024; Song et al., 2010), reflects diminished physiological reserve and resilience to stressors (Bergman et al., 2009; Melson et al., 2024). Hypoglycemia and frailty have a bidirectional relationship: hypoglycemia can lead to disability, cognitive decline, and frailty, while frailty increases hypoglycemia risk through factors such as polypharmacy, comorbidities, and functional limitations (Abdelhafiz et al., 2016; Melson et al., 2024).

Although self-efficacy, hypoglycemia, and frailty are each recognized as important in diabetes management, their interrelationships in older adults with type 2 diabetes remain understudied (Al-Khawaldeh et al., 2012; Çalli, 2021; Kirkman et al., 2012; Krichbaum et al., 2003; Norris et al., 2001). Understanding these connections can inform targeted interventions to strengthen self-efficacy, reduce hypoglycemia risk, and mitigate frailty, ultimately improving health outcomes and quality of life in this population.

### Significance of the study

The growing prevalence of T2DM among older adults represents a critical public health challenge due to its association with multiple comorbidities, functional impairments, and geriatric syndromes. Despite advances in care, older adults remain particularly vulnerable to hypoglycemia, frailty, and reduced self-management capacity, which collectively contribute to diminished quality of life and increased healthcare utilization. While self-efficacy is a well-established determinant of self-management, its relationship with fear of hypoglycemia and frailty in this population is poorly understood. Investigating these interrelationships can address a significant gap in the literature and guide the development of targeted interventions to enhance self-efficacy, alleviate psychological barriers, and reduce physical vulnerability—ultimately improving health outcomes and promoting healthy aging in older adults with T2DM.

## Objective

This study aimed to examine the influence of fear of hypoglycemia and frailty on self-efficacy in diabetes management among older adults diagnosed with type 2 diabetes mellitus (T2DM). Specifically, the study sought to determine the associations between these factors and identify significant predictors of self-efficacy, accounting for relevant socio-demographic and clinical characteristics.

## Methods

### Study design and setting

This cross-sectional descriptive study was conducted following the Strengthening the Reporting of Observational Studies in Epidemiology (STROBE) guidelines. Data were collected from health insurance diabetic outpatient clinics. These specialized clinics provide comprehensive care and management for individuals with diabetes and operate under the national health insurance system, ensuring access to essential medical services at affordable costs.

### Sample size and study population

Participants were recruited according to predefined inclusion and exclusion criteria. Eligible participants were older adults aged 60 years or older with a confirmed medical diagnosis of type 2 diabetes mellitus for at least 6 months prior to data collection. Participants were required to be able to communicate effectively, understand the study objectives, and provide written informed consent.

Individuals were excluded if they had severe cognitive impairment, diagnosed psychiatric disorders, acute medical conditions, or sensory or communication impairments that could interfere with accurate completion of the study questionnaires. Participants with incomplete data were also excluded from the final analysis.

The sample size was determined using G*Power, a widely accepted statistical software for power analysis (Faul et al., 2007). Parameters for the calculation included an effect size of 0.1 (small-to-medium according to Cohen’s guidelines), an alpha level of 0.05, a power of 0.90, and 16 predictors (including demographic and predictor variables). This yielded a required sample size of at least 300 participants. To account for potential nonresponse, the sample size was increased to 320. Following the exclusion of nine ineligible patients and 11 who declined participation, the final sample consisted of 300 participants. A convenience sampling method was employed, recruiting participants from the previously mentioned diabetic clinics.

### Instruments

#### Demographic questionnaire

It includes socio-demographic data such as age, sex, marital status, residence, family type, employment status, and income. Additionally, lifestyle factors are evaluated, such as smoking behavior and exercise. Additionally, it delves into medical history, including diabetes duration, family history of diabetes mellitus, treatment regimen, and complications of diabetes mellitus. Participants with lesser incomes—those making less than 6,000 EGP a month— National economic classifications were used to determine the threshold of 6,000 EGP, which is below the average monthly household income in Egypt (Central Agency for Public Mobilization and Statistics, 2020).

### Fear of hypoglycemia screener

The Fear of Hypoglycemia Screener, developed by Liu et al. (2023), assesses the level of fear associated with hypoglycemia (Liu et al. 2023). It comprises nine items across two domains: worry (6 items) and avoidance behavior (3 items). Each item is rated on a five-point Likert scale ranging from *strongly disagree* (1) to *strongly agree* (5), yielding a total possible score ranging from 9 to 45, with higher total scores indicating greater fear of hypoglycemia.

Regarding validity and reliability, the original version underwent Exploratory Factor Analysis (EFA), which identified a single-factor structure explaining 66% of the variance, indicating good construct validity. Confirmatory Factor Analysis (CFA) confirmed this one-factor model, with satisfactory fit indices (CFI = 0.95, TLI = 0.94, RMSEA = 0.06). Internal consistency was high (Cronbach’s *α* = 0.88).

In the present study, the scale demonstrated even higher internal consistency (Cronbach’s α = 0.97).

### Chinese frailty screening scale (CFSS-10)

The 10-item Chinese Frailty Screening Scale (CFSS-10), developed by Ye et al. (2022), is a comprehensive tool for assessing frailty in older adults Ye et al. (2022). It evaluates various aspects of physical and mental health through a series of questions. The first item addresses the presence of at least five diagnosed illnesses, such as hypertension, diabetes, cancer, chronic lung disease, and heart disease. Other items assess exhaustion, loss of appetite, visual impairment, hearing loss, and reduced physical resistance (e.g., difficulty climbing stairs without resting). The scale also evaluates physical inactivity, attention deficits, orientation problems, and depressive symptoms.

The CFSS-10 includes 10 dichotomous (yes/no) items, resulting in a total score range from 0 to 10, with higher scores reflecting greater levels of frailty. In the present study, the CFSS-10 demonstrated excellent internal consistency (Cronbach’s *α* = 0.93).

### The diabetes management self-efficacy scale

The Diabetes Management Self-Efficacy Scale, developed by Bijl et al. (1999), measures self-efficacy in managing T2DM Bijl et al. (1999). It consists of 20 items across four subscales: nutrition-specific and weight (5 items), nutrition-general and medical treatment (9 items), physical exercise (3 items), and blood sugar management (3 items). Each item is rated on a five-point Likert scale, ranging from 1 (not at all confident) to 5 (very confident). Total scores range from 20 to 100, with higher scores indicating greater confidence in diabetes self-management.

The scale has demonstrated strong psychometric properties, with a Cronbach’s *α* of 0.81 and test–retest reliability of *r* = 0.79. Exploratory Factor Analysis (EFA) has shown good construct validity (KMO > 0.80; Bartlett’s test, *p* < 0.001), while Confirmatory Factor Analysis (CFA) indicated good model fit (CFI and TLI > 0.90; RMSEA < 0.08). In the present study, the scale exhibited even higher internal consistency (Cronbach’s *α* = 0.95).

### Study procedures

#### Tool preparation and pilot study

The research instruments, Fear of Hypoglycemia Screener, CFSS-10, and Diabetes Management Self-Efficacy Scale, were translated into Arabic with meticulous attention to accuracy and cultural appropriateness. Bilingual experts fluent in English and Arabic conducted the translations, followed by back-translation into English to verify linguistic equivalence and resolve any discrepancies. Face validity was assessed by expert panels to ensure the translated tools effectively measured the intended constructs in the Arabic context. Feedback from potential participants was obtained to confirm item clarity, relevance, and cultural suitability. Reliability was examined using Cronbach’s α to assess internal consistency. A pilot study was conducted with 30 older adults with T2DM to evaluate clarity, relevance, and reliability. These participants were excluded from the main study. The pilot results indicated no modifications were necessary for the instruments.

### Data collection

Data collection was conducted from September to December 2024 in the waiting areas of diabetic clinics. The process began with a comprehensive orientation in which the study’s purpose was explained, the voluntary nature of participation was emphasized, and confidentiality measures were outlined to foster trust. Participants were encouraged to ask questions, which were addressed promptly. Trained researchers facilitated data collection and obtained written informed consent from each participant before participation. To maintain anonymity, participants were instructed not to include any personal identifiers on the questionnaire. Participation was entirely voluntary, and individuals could decline simply by not completing the questionnaire. Completing the questionnaire took approximately 15–20 min. Upon completion, participants sealed their responses in envelopes and placed them in a secure collection box to ensure privacy and confidentiality.

### Data analysis

Data were analyzed using IBM SPSS Statistics, Version 27 (IBM Corp., Armonk, NY, USA). Relationships between the dependent and independent variables were examined using independent-sample t-tests and, when appropriate, one-way analysis of variance (ANOVA). Pearson’s correlation coefficient was used to assess correlations among study variables. The regression assumptions were examined prior to conducting the multiple linear regression analysis. Normality was assessed by inspecting histograms and normal probability (P–P) plots of the standardized residuals, which indicated an approximately normal distribution. Multicollinearity was assessed using Tolerance and Variance Inflation Factor (VIF) statistics. All tolerance values were above the 0.10 threshold (ranging from 0.20 to 0.93), and all VIF values were below the commonly used cutoff of 5 (ranging from 1.19 to 4.59), indicating that multicollinearity did not pose a threat to the stability of the regression model (Hair et al., 2013; Kim, 2019). Independence of residuals was assessed using the Durbin–Watson statistic, which was 1.71, supporting the assumption of residual independence. These diagnostics confirmed that the data met the assumptions required for multiple linear regression analysis. Multiple linear regression analysis was performed to identify significant predictors of self-efficacy in diabetes management. Statistical significance was set at *p* < 0.05.

## Results

### Associations between socio-demographic and clinical characteristics, fear of hypoglycemia, frailty, and diabetes management self-efficacy

Table 1 shows the associations between socio-demographic and clinical characteristics and diabetes management self-efficacy. No significant associations were observed with age, sex, marital status, education level, residence, or diabetes duration (*p* > 0.05). In contrast, self-efficacy differed significantly by Employment status (*p* = 0.004), with higher scores observed among retired participants. Significant associations were also found for smoking status (*p* < 0.001) and family history of diabetes (*p* < 0.001). Diabetes management self-efficacy varied significantly according to treatment regimen (*p* < 0.001), comorbidities (*p* < 0.001), and diabetes-related complications (*p* = 0.003).

**Table 1:** Relationship between socio-demographic and diabetes management self-efficacy (*N* = 300).

Variables	*n*	%	Diabetes management self-efficacy	t/f (*p*-value)
Age				
<65	94	31.3	78.20 ± 13.03	2.40 (0.092)
65–69	87	29.0	80.25 ± 10.18	
>70	119	39.7	81.66 ± 10.94	
Sex				
Male	168	56.0	78.81 ± 9.59	1.813 (0.071)
Female	132	44.0	81.23 ± 12.71	
Marital status				
Single	4	1.3	90.75 ± 3.20	2.097 (0.125)
Married	222	74.0	79.69 ± 11.54	
Divorced/widow	74	24.7	81.01 ± 11.37	
Education level				
Illiterate	128	42.7	79.92 ± 10.39	0.085 (0.968)
Primary	76	25.3	80.69 ± 11.64	
Secondary	75	25.0	80.18 ± 12.11	
University	21	7.0	79.66 ± 15.34	
Residence				
Urban	149	49.7	80.87 ± 12.52	1.052 (0.294)
Rural	151	50.3	79.47 ± 10.37	
Employment status				
Unemployed	94	31.3	78.64 ± 11.04	5.739 (0.004)
Employed	107	35.7	78.58 ± 11.45	
Retired	99	33.0	83.32 ± 11.41	
Income				
Not enough	41	13.7	77.48 ± 11.24	−1.613 (0.107)
Enough	259	86.3	80.59 ± 11.49	
Smoking				
Yes	97	32.3	84.47 ± 11.51	0.189 (<0.001)
No	203	67.7	78.11 ± 10.92	
DM duration				
Less than 5 years	39	13.0	78.92 ± 14.00	2.241 (0.108)
5–10	112	37.3	78.73 ± 10.48	
More than 10	149	49.7	81.57 ± 11.40	
Family history of diabetes				
Yes	203	67.7	81.84 ± 12.14	3.724 (<0.001)
No	97	32.3	76.67 ± 9.10	
DM treatment regimens				
Diet and exercise	16	5.3	84.31 ± 8.31	12.499 (<0.001)
Oral	168	56.0	77.26 ± 11.02	
Insulin	36	12.0	79.00 ± 9.68	
Insulin and oral	80	26.7	85.97 ± 11.49	
Comorbidities				
HTN	95	31.7	82.61 ± 9.70	5.029 (<0.001)
HD	139	46.3	81.07 ± 12.95	
Anemia	31	10.3	77.67 ± 7.76	
Renal	22	7.3	72.04 ± 6.67	
Respiratory	3	1.0	72.33 ± 1.15	
Bone	10	3.3	72.40 ± 13.47	
DM complications				
Neuropathy	64	21.3	77.75 ± 10.89	4.742 (0.003)
HD	94	31.3	77.90 ± 13.53	
Retinopathy	100	33.3	82.81 ± 9.91	
Diabetic foot infection	42	14.0	82.64 ± 9.14	
Neuropathy	64	21.3	77.75 ± 10.89	

t, independent *t*-test. f, ANOVA test.

### Descriptive statistics of study variables

Table 2 presents the means and standard deviations of the primary study variables. The mean scores for the Fear of Hypoglycemia Screener, Frailty Screening, and Diabetes Management Self-Efficacy were 36.13 ± 7.70, 2.93 ± 2.28, and 80.17 ± 11.49, respectively.

**Table 2:** Mean of fear of hypoglycemia screener, frailty screening, and diabetes management self-efficacy (*n* = 300).

Variables	Possible total score range	Minimum	Maximum	Mean	Std. deviation
Fear	9–45	10.00	45.00	36.13	7.70
Frailty	0–10	0	10.00	2.93	2.28
DM management self-efficacy	20–100	23.00	100.00	80.17	11.49
Nutrition-specific and weight	5–25	7.00	25.00	19.95	3.33
Nutrition general and medical treatment	9–45	9.00	45.00	36.10	5.15
Physical exercise	3–15	3.00	15.00	11.36	2.44
Blood sugar	3–15	3.00	15.00	12.75	1.69

### Correlation between study variables

Table 3 presents the correlations among the primary study variables. A significant weak positive correlation was found between fear of hypoglycemia and frailty (*r* = 0.277, *p* < 0.01). In contrast, fear of hypoglycemia demonstrated a weak negative correlation with diabetes management self-efficacy (*r* = −0.270, *p* < 0.01). Frailty demonstrated a moderate negative association with diabetes management self-efficacy (*r* = −0.464, *p* < 0.01). Detailed correlations between the subscales are also provided in Table 3.

**Table 3:** Correlation between fear of hypoglycemia screener, frailty screening, and DM management self-efficacy (*n* = 300).

Variables	1	2	3	4	5	6	7
1. Fear of hypoglycemia	1						
2. Frailty	0.277**	1					
3. The diabetes management self-efficacy	−0.270**	−0.464**	1				
4. Nutrition-specific and weight	−0.284**	−0.334**	0.783**	1			
5. Nutrition general and medical treatment	−0.282**	−0.362**	0.783**	0.770**	1		
6. Physical exercise	−0.220**	−0.306**	0.659**	0.773**	0.649**	1	
7. Blood sugar	−0.298**	−0.406**	0.905**	0.954**	0.881**	0.824**	1

**Correlation is significant at the 0.01 level (two-tailed).

### Multiple linear regression analysis

Table 4 demonstrates that several factors independently influenced self-efficacy in diabetes management among older adults with type 2 diabetes. Non-smoking status was significantly associated with lower self-efficacy compared with smokers (*β* = −0.18; 95% CI: −7.39 to −1.84; *p* = 0.001). Among comorbidities, renal disease (*β* = −0.14; 95% CI: −10.92 to −1.46; *p* = 0.010) and bone disorders (*β* = −0.10; 95% CI: −13.60 to −0.10; *p* = 0.046) were significant negative predictors of self-efficacy. Psychological and functional factors showed the strongest effects, with higher fear scores (*β* = −0.14; 95% CI: −0.37 to −0.05; *p* = 0.008) and greater frailty (*β* = −0.26; 95% CI: −1.91 to −0.76; *p* = 0.001) associated with substantial reductions in self-efficacy. Employment status, family history of diabetes, treatment regimens, and most diabetes complications were not significantly associated with self-efficacy. Overall, the model explained 32.1% of the variance in self-efficacy (adjusted *R*^2^ = 0.280; *p* < 0.001), and multicollinearity was not a concern, as tolerance values were acceptable and all VIFs were below 5.

**Table 4:** Factors affecting self-efficacy in diabetes management among type 2 diabetic older adults.

Factors	*B*	SE	*β*	*t*	CI (95%)	Sig	Tolerance	VIF
Job								
Unemployed		Reference						
Employed	−1.91	1.56	−0.080	−1.22	−4.98 to 1.16	0.222	0.56	1.76
Retired	−1.38	1.58	−0.05	−0.87	−4.50 to 1.72	0.382	0.57	1.74
Smoking								
Yes		Reference						
No	−4.62	1.41	−0.18	−3.27	−7.39 to −1.84	0.001	0.72	1.37
Family history of diabetes								
Yes		Reference						
No	−1.01	1.35	−0.04	−0.75	−3.68 to 1.64	0.453	0.79	1.26
DM treatment Regimens								
Diet and exercise		Reference						
Oral	−4.47	2.63	−0.19	−1.69	−9.66 to 0.72	0.091	0.18	4.59
Insulin	−2.69	3.06	−0.07	−0.87	−8.73 to 3.34	0.380	0.31	3.13
Insulin and oral	−1.21	2.78	−0.047	−0.43	−6.69 to 4.26	0.662	0.20	4.78
Comorbidities								
HTN		Reference						
HD	−0.82	1.37	−0.03	−0.60	−3.52 to 1.87	0.547	0.67	1.47
Anemia	−3.64	2.07	−0.09	−1.75	−7.72 to 0.43	0.080	0.79	1.25
Renal	−6.19	2.40	−0.14	−2.57	−10.92 to −1.46	0.010	0.80	1.23
Respiratory	−6.02	5.84	−0.05	−1.03	−17.52 to 5.47	0.304	0.93	1.06
Bone	−6.85	3.42	−0.10	−2.00	−13.60 to −0.10	0.046	0.83	1.19
DM complications								
Neuropathy		Reference						
HD	0.80	1.75	0.03	0.45	−2.64 to 4.25	0.646	0.48	2.08
Retinopathy	2.69	1.65	0.11	1.63	−0.55 to 5.95	0.104	0.52	1.91
Diabetic foot infection	2.85	1.99	0.08	1.43	−1.07 to 6.77	0.153	0.66	1.50
Fear	−0.21	0.08	−0.14	−2.66	−0.37 to −0.05	0.008	0.83	1.19
Frailty	−1.34	0.29	−0.26	−4.60	−1.91 to −0.76	0.001	0.71	1.39

R2 = 0.321, Adjusted R2 = 0.280, *p* < 0.001, B = Unstandardized coefficient; SE = Standard error; β = Standardized coefficient; t = t-test statistic; CI = Confidence interval, VIF = variance inflation factor.

## Discussion

Managing type 2 diabetes in older adults is challenging due to the combined effects of fear of hypoglycemia, frailty, and self-efficacy. In later life, day-to-day self-management depends not only on knowledge and access to care, but also on psychological safety (fear of hypoglycemia), functional capacity (frailty), and the confidence to carry out complex behaviors (self-efficacy). Fear of hypoglycemia undermines confidence in treatment adherence, while frailty exacerbates physical and cognitive barriers. This behavioral avoidance increases the long-term risks of hyperglycemia-related complications (Fariba et al., 2024). Frailty also reduces physiological reserve and heightens vulnerability to stressors, which can further limit the feasibility of routine diabetes self-care (Robertson et al., 2023). Self-efficacy plays a key role in addressing these challenges. In the present study, fear of hypoglycemia and frailty were related, and both were associated with lower diabetes management self-efficacy, with frailty showing the comparatively stronger inverse relationship. This analysis highlights the interconnected impact of these factors on self-management of diabetes and emphasizes the need for holistic, personalized care to overcome barriers and improve outcomes for this vulnerable group (Esferjani et al., 2022; Randväli et al., 2024).

The findings of this study provide an understanding of the dynamic interactions among fear of hypoglycemia, frailty, and self-efficacy in diabetes management among older adults with type 2 diabetes mellitus (Zabeeri, 2025). These variables do not operate in isolation but rather influence one another, creating a complex network of psychological, physical, and behavioral challenges that impede optimal diabetes management in this vulnerable demographic. The interplay between psychological factors, such as hypoglycemia anxiety, and physical vulnerabilities like frailty is well-established. Studies by Hendriks et al. (2019) demonstrate how fear of hypoglycemia predicts poorer engagement in self-care practices, while Qi et al. (2023) describe frailty as a state of heightened vulnerability to even minor stressors (Hendriks et al., 2019; Qi et al., 2023). Together, these patterns support a clinically meaningful “psychological–functional” pathway: when hypoglycemia is anticipated as dangerous or uncontrollable, and when physical reserves are reduced, older adults may become less confident in their ability to implement diet, activity, monitoring, and medication behaviors consistently.

The study underscores the influence of fear of hypoglycemia on self-efficacy in diabetes management. Research by Cryer (2005) shows that hypoglycemia-associated autonomic failure worsens with aging, compounding the physiological and psychological burdens for older adults (Cryer, 2005). This aligns with findings from Graveling and Frier (2010a), who reported that repeated hypoglycemic episodes reduce confidence in glucose management (Graveling and Frier, 2010a). Fear of hypoglycemia, a psychological construct deeply tied to the lived experience of individuals managing diabetes, emerged as a determinant of lower self-efficacy (Abdelhafiz et al., 2015). This finding aligns with prior research that documents the detrimental effects of hypoglycemia anxiety on individuals’ confidence to engage in self-management behaviors such as medication adherence, blood glucose monitoring, and dietary regulation (Escudero et al., 2024; Martín-Timón and Del Cañizo-Gómez, 2015; Surrati et al., 2023). Fear is often grounded in prior symptomatic episodes, worries about nocturnal events, and concerns about falls, injury, or needing assistance concerns that may intensify with aging, comorbidity, and reduced physiological reserve. Consequently, individuals often develop avoidance behaviors, such as restricting insulin use or overcompensating with carbohydrate intake, mitigating perceived risks and inadvertently undermining glycemic control (Umegaki, 2024; Zeng et al., 2023). Clinically, reduced confidence may act as a “behavioral bridge” through which fear translates into less consistent self-management, potentially increasing glycemic instability and perpetuating fear.

Moreover, the findings suggest that fear of hypoglycemia is not merely a psychological burden but also a behavioral and physical risk factor, closely intertwined with frailty. The positive relationship between fear of hypoglycemia and frailty highlights a bidirectional pattern wherein fear-induced behaviors, such as reduced physical activity, may contribute to muscle weakness and functional decline, while frailty may exacerbate fear by increasing the perceived consequences of hypoglycemic episodes (Abdelhafiz et al., 2015). The reciprocal effects of frailty and fear have been supported by Prasad-Reddy et al. (2022), who noted that frailty amplifies the adverse outcomes of poor glycemic control due to reduced functional reserves (Prasad-Reddy et al., 2022). Although the association was not large, it remains clinically important in later life because even modest reductions in activity can accelerate deconditioning and functional loss. This cyclical relationship underscores the importance of addressing fear and frailty simultaneously in interventions to restore confidence and functionality in older adults with diabetes (Li et al., 2022).

Frailty itself emerged as a significant determinant of lower self-efficacy in diabetes management, further validating its central role in shaping the physical and psychological challenges faced by older adults. As per Strain et al. (2021), frailty diminishes physical reserves and complicates the execution of self-care tasks, such as exercise or meal preparation (Strain et al., 2021). These findings mirror those of Almomani et al. (2021), who reported a direct link between frailty and poorer glycemic control (Almomani et al., 2021). Frailty is characterized by a decline in physiological reserves, leaving individuals less capable of managing physical and psychological stressors, including the complexities of diabetes self-care. When functional capacity declines, diabetes tasks that appear straightforward in standard education models meal planning, safe physical activity, medication organization, glucose monitoring, and response to symptoms can become cognitively and physically taxing, eroding perceived control and confidence (Freeman, 2019). By identifying frailty as a key determinant of self-efficacy, the study underscores the necessity of incorporating frailty assessments into routine diabetes care protocols to identify and support at-risk individuals (Randväli et al., 2024).

The observed negative relationship between frailty and self-efficacy highlights the profound impact of physical limitations on individuals’ confidence to manage their condition effectively. (Çalli et al., 2021) emphasizes the significance of self-efficacy in care for diabetes, noting that interventions targeting self-efficacy can improve adherence (Çalli, 2021). Self-efficacy, conceptualized by Bandura (1986) as the belief in one’s ability to execute behaviors necessary to achieve specific outcomes, is a critical mediator of diabetes self-management behaviors (Bandura, 1986). Higher self-efficacy is consistently associated with better adherence to treatment regimens, improved glycemic control, and enhanced overall wellbeing (Çalli, 2021; Jiang et al., 2019). However, frailty undermines this confidence by imposing physical and cognitive barriers that erode individuals perceived control over their health. This supports interventions that do more than provide information—interventions should actively reduce functional barriers and create achievable “mastery experiences” that rebuild confidence (Bandura, 1997; Vaccaro et al., 2019).

The study also reveals the influence of socio-demographic factors on self-efficacy, further contextualizing the challenges faced by older adults in managing diabetes. Income disparities, as described by Grintsova et al. (2014), highlight that low-income individuals often lack access to preventive care and diabetes education programs, exacerbating inequalities in self-management outcomes (Graveling and Frier, 2010b). Employment status, income level, and smoking behavior emerged as predictors of self-efficacy, reflecting the broader social and economic determinants of health (Strain et al., 2021). For example, retired individuals and homemakers demonstrated higher self-efficacy compared to their unemployed counterparts, possibly due to greater stability in daily routines, access to social support networks, or fewer competing stressors. Income level also played a role, with higher-income individuals exhibiting better self-efficacy, likely due to greater access to healthcare resources, educational opportunities, and nutritious food options (Zabeeri, 2025). Notably, non-smokers rather than smokers showed lower self-efficacy. This counterintuitive pattern should be interpreted cautiously, as it may reflect residual confounding or selection effects, such as illness-driven smoking cessation, differences in functional status, or reporting tendencies, rather than any protective role of smoking. Accordingly, this finding warrants further investigation using designs that more robustly account for health trajectories and detailed smoking history.

Treatment regimens and comorbidities further shaped self-efficacy outcomes in this study, highlighting the complex interplay of medical and behavioral factors in diabetes management. Participants using both insulin and oral medications reported higher self-efficacy compared to those relying solely on lifestyle modifications or oral therapies (Park et al., 2024; Prasad-Reddy et al., 2022). However, in the multivariable analysis, treatment regimens did not remain independently associated with self-efficacy, suggesting that unadjusted differences may reflect disease severity, monitoring exposure, or related factors rather than a direct regimen effect. Comorbidities, such as renal and bone diseases, were associated with lower self-efficacy, underscoring the additional burden these conditions impose on individuals already managing diabetes (Umegaki, 2024; Vaccaro et al., 2019). These comorbidity associations are clinically coherent: renal disease can heighten hypoglycemia risk through altered drug clearance and nutritional variability, while bone disorders may restrict mobility and increase fear of falls each potentially undermining confidence and willingness to engage in activity and other self-care behaviors.

Rather than implying deterministic effects, the observed magnitudes are better understood as risk signals within a multifactorial system of self-management behavior. Consistent with this, the multivariable model explained only a limited share of the variability in self-efficacy, indicating substantial unexplained variance and reinforcing that diabetes self-management confidence is inherently multifactorial (Xu et al., 2018). Additional determinants likely include health literacy, cognitive status, depressive symptoms, diabetes distress, prior severe hypoglycemia, perceived social support, regimen complexity and polypharmacy, mobility limitations, access barriers, and the quality of patient provider communication (Jafari et al., 2024; Young-Hyman et al., 2016).

Taken together, the findings offer actionable implications for clinical care, particularly around fear reduction and frailty prevention. The interplay between fear of hypoglycemia, frailty, and self-efficacy underscores the need for holistic and personalized diabetes care (American Diabetes Association Professional Practice, 2023). First, fear of hypoglycemia should be proactively identified and addressed as a modifiable barrier to confidence and engagement, not merely as a symptom of anxiety. Practical steps include structured conversations about prior hypoglycemia, symptom recognition, fear-driven behaviors (e.g., intentional under-dosing, over-snacking, avoidance of activity), and individualized action plans. Interventions should address hypoglycemia fear through counseling and other supportive approaches to reduce anxiety, alleviate avoidance behaviors, and restore confidence in self-management (Melson et al., 2025; Strain et al., 2021). Where feasible, prioritizing safer regimens (lower hypoglycemia risk), simplifying complex plans, and using supportive monitoring strategies can reduce uncertainty and improve psychological safety, which may translate into stronger self-efficacy (LeRoith et al., 2019).

Simultaneously, frailty-focused approaches, including tailored exercise programs, nutritional support, and physical rehabilitation, can enhance functional capacity and mitigate physical barriers (Sirikul et al., 2024; Weinstock et al., 2024). Because frailty showed a stronger relationship with self-efficacy, preserving mobility and strength may yield disproportionate gains in confidence and sustained self-management. Multicomponent interventions (resistance and balance training, nutrition optimization, fall risk assessment, and rehabilitation referral when indicated) may help prevent the downward spiral in which weakness increases fear, fear reduces activity, and inactivity accelerates frailty (Bilici and Kılıç, 2025; Prasad-Reddy et al., 2022).

Diabetes education is equally critical, empowering older adults by addressing physical limitations, psychological burdens, and socio-economic disparities. Tailored programs, subsidized healthcare, and accessible resources for underserved populations can bridge knowledge gaps and strengthen self-efficacy (Allen et al., 2022; American Diabetes Association Professional Practice, 2023; Escudero et al., 2024). Finally, education that improves self-efficacy in older adults should be practical and adaptive using simplified routines, teach-back techniques, caregiver engagement when appropriate, and individualized goals anchored in functional status and patient priorities (American Diabetes Association Professional Practice Committee, 2024).

### Strengths and limitations

This study has several notable strengths. It employed a rigorous methodological approach, utilizing well-established and validated instruments to assess key constructs related to diabetes management, fear of hypoglycemia, frailty, and self-efficacy. The relatively large sample size (*n* = 300), determined through an *a priori* power analysis, provided sufficient statistical power to detect meaningful associations among the study variables. In addition, the application of multiple statistical techniques, including multiple linear regression analysis, allowed for a robust examination of the associative relationships between psychological and geriatric factors and diabetes management self-efficacy among older adults.

Nevertheless, several limitations should be acknowledged. First, the use of a convenience sampling method may limit the generalizability of the findings to the broader population of older adults with type 2 diabetes mellitus and may introduce selection bias. Second, the reliance on self-reported measures raises the possibility of response bias, including social desirability bias, particularly when participants responded to sensitive psychosocial items. Importantly, due to the cross-sectional design of the study, causal inferences cannot be drawn, and the observed relationships should be interpreted as associations rather than cause–effect relationships. These limitations should be considered when interpreting the results. Future research employing probability-based sampling methods, objective clinical indicators, and longitudinal or experimental designs is recommended to enhance external validity and support stronger causal inference.

## Conclusion

This study demonstrates that fear of hypoglycemia significantly impacts frailty and diabetes management self-efficacy, with frailty potentially mediating this relationship. Socio-demographic factors, including age, education, and duration of diabetes, also play a pivotal role in shaping these outcomes. These findings highlight the need for tailored interventions that address both psychological and physical factors to enhance diabetes self-management among older adults with T2DM. Integrating psychological support and frailty assessment into routine diabetes care, alongside patient-centered education and support systems, may effectively improve self-efficacy and overall disease management in this population.

### Relevance for clinical practice

The findings underscore the importance of multifaceted, patient-centered approaches that address psychological, physical, and social dimensions to optimize diabetes care and improve outcomes for older adults. Future research should focus on developing and evaluating intervention strategies aimed at reducing identified barriers and enhancing self-efficacy in diabetes management. Healthcare professionals should prioritize comprehensive education and psychological support to alleviate hypoglycemia-related fears and promote effective diabetes self-management. Incorporating frailty screening and management into routine care for older adults with T2DM is essential to support their engagement in self-care behaviors. Additionally, tailored interventions that account for demographic factors, such as employment status, may further strengthen patients’ confidence and capacity to manage their condition effectively.

## Data Availability

The original contributions presented in the study are included in the article/supplementary material, further inquiries can be directed to the corresponding author/s.
